# Understanding the Dark Side of Online Reviews on Consumers' Purchase Intentions in E-Commerce: Evidence From a Consumer Experiment in China

**DOI:** 10.3389/fpsyg.2021.741065

**Published:** 2021-10-15

**Authors:** Junbin Wang, Xiaojun Fan, Xiangdong Shen, Yurong Gao

**Affiliations:** ^1^Business School, Changshu Institute of Technology, Changshu, China; ^2^School of Management, Fudan University, Shanghai, China; ^3^School of Management, Shanghai University, Shanghai, China

**Keywords:** consumer experiment, online reviews, information inconsistency, product type, purchase intention, online word-of-mouth

## Abstract

**Background:** Online review, as an important way of electronic word-of-mouth (eWOM) communication, plays an important role in e-commerce. However, few studies have examined the dark side of online reviews and their effect on consumers' purchase intentions. Information inconsistency is one of the dark sides that plays a critical role in influencing consumers' purchase intentions through online reviews.

**Methods:** Using a 2^*^2 between-subject design that explores the main effects of the type of information inconsistency (vertical- vs. horizontal-attribute inconsistency) on purchase intention and the moderating effect of product type (search vs. experience product).

**Results:** This study examines whether and how the type of information inconsistency between online recommendations and reviews influences consumer purchase decision-making.

**Conclusions:** The findings show that vertical-attribute inconsistency leads to a lower purchase intention for search products; moreover, both vertical- and horizontal-attribute inconsistencies have no significant effect on purchase intention for experience products.

## Introduction

With the advent of the “Internet+” era, online consumption patterns (e.g., online booking and shopping) have become increasingly popular. Moreover, the prevalence of social media has made the interaction among consumers more convenient (Hall, 2018) and enriched the modes of sharing consumer reviews (Yan et al., 2016), further promoting e-commerce and online word-of-mouth (WOM). Previous research has pointed out that online WOM influences consumers' purchase intention (Seiler et al., 2017; Wu and Lin, 2017; Chen and Farn, 2020). Online recommendations and reviews are the two most important forms of WOM, and they are highly influential in promoting online shopping consumption (Schmitt et al., 2011). Online recommendations are generated through referral systems (e.g., personal-recommendation websites), giving an explicit suggestion for consumers (Azer and Alexander, 2020) that are an effective tool for consumers to discover items they may be interested in from a large collection of products. They help consumers reduce search costs and avoid being overwhelmed by the breadth of available products (Baum and Spann, 2014; Hong et al., 2017; Adomavicius et al., 2018). Meanwhile, online reviews play an important role in consumers' decision-making (Cheung et al., 2008; Choi et al., 2020), as they provide information from reviewers who actively discover quality information (Huang et al., 2015) and share direct experience (Mudambi and Schuff, 2010), thereby reducing consumers' uncertainty (Elwalda et al., 2016; Liang, 2016; Lantzy and Anderson, 2020). Moreover, consumers regard online reviews as a basis for deciding whether to purchase products recommended by the referral systems of e-commerce websites or friends in social media (Zhang et al., 2014). Undoubtedly, online recommendations and reviews interactively affect consumers' purchase intention, but existing literature that jointly research these two avenues for online WOM is sparse (Baum and Spann, 2014; Jabr and Zheng, 2014). However, it is very important for managers to better understand the impact of the type of information inconsistency on consumers' purchase intention and how to reduce inconsistency and increase consumers' purchase intention.

Existing literature related to online WOM mainly focus on three aspects: (1) online WOM and marketing performance (Rosario et al., 2016), (2) online WOM and management (Eelen et al., 2017) and (3) online WOM and consumers' decision-making (Yan et al., 2016). However, these studies investigate online recommendations and reviews separately, ignoring the interaction between them (Baum and Spann, 2014). As consumers tend to search for information from multiple sources (Ma et al., 2017) and their purchase decisions depend on information consistency (Quaschning et al., 2015), online recommendations and reviews (as the main sources of product information) need to be studied simultaneously. Consumers find products that meet their needs based on network recommendations and decide whether to buy the product based on online reviews. The consistency or inconsistency of information from both sources undoubtedly affects consumers' purchase decision-making behavior.

Furthermore, previous literature on information inconsistency, although few, mainly researched the relationship between purchase intention and the degree of inconsistency (De Maeyer, 2012) or the effect of inconsistency between review valence and review score on sales (Eslami and Ghasemaghaei, 2018; Zheng et al., 2018). Meanwhile, this present study focuses on whether and how the type of information inconsistency influences consumers' perceived uncertainty and purchase intention. Specifically, in terms of product attributes, this study divides the type of information inconsistency between online recommendations and reviews into vertical- and horizontal-attribute inconsistencies. Because, for different types of products, consumers' perception of product quality through product information is different (Nelson, 1970), for different products, the perception of product uncertainty is different due to the inconsistency between online recommendations and reviews. Moreover, this study investigates the moderating role of the product type on the effects of information inconsistency (between online recommendations and reviews) on consumers' perceived uncertainty and purchase intention. Specifically, the main research questions of this study are as follows:

(1) What kind of product-attribute information from online recommendations can affect consumers more (vertical or horizontal attribute)? How does information inconsistency between online recommendations and reviews affect consumers' perceived uncertainty and purchase intention?
(2) How do the moderating effect and boundary condition of product type (search product, experience product) impact the relationship between information inconsistency and consumers' perceived uncertainty and purchase intention?

In addressing these questions, a quasi-experiment was conducted to test the main effect of the type of information inconsistency between online recommendations and reviews on purchase intention, the mediating role of perceived uncertainty, and the moderating role and boundary condition of product type in the abovementioned relationships.

This paper consists of five parts. The theoretical model and hypothesis development of this study is presented in the section Theoretical Model and Hypothesis Development following by the introduction, while the contexts of the third section are the Research Methodology. Then, data analysis results are analyzed in detail in the section Results. In the last section, the findings and highlights of the theoretical and practical implications for researchers and companies will be discussed as well as some suggestions for future research according to the limitations of this study.

## Theoretical model and hypothesis development

### TRA and TPB Theories

The theory of reasoned action (TRA) was proposed by American scholars Fishbein and Ajzen in 1975 (Fishbein and Ajzen, 1975). It is mainly used to analyze how attitudes consciously affect individual behavior and pay attention to the process of attitude formation based on cognitive information. The theory holds that individual behavior can be reasonably inferred from behavior intention to some extent, and individual behavior intention is determined by behavior attitude and subjective criteria.

The theory of planned behavior (TPB) was proposed by Ajzen (1991). This theory is evolved on the basis of the theory of rationed action (TRA), which is mainly used to predict and understand human behavior. Because TRA assumes that the individual is completely voluntary to control whether to adopt a specific behavior, it ignores the ethical decisions made by the core users, especially the individual characteristics. So Ajzen (1991) added a third element: cognitive behavioral control. This study takes the type of information inconsistency as an independent variable, perceived uncertainty as a mediation variable and purchase intention as a dependent variable. When consumers perceived uncertainty is stronger, individual perceptual control will weaker, which will weaken the purchase intention.

### Type of Information Inconsistency

Consumers evaluate multiple cues simultaneously for their purchase decision-making (Kim and Tanford, 2019). Their online behavior goes through five stages: attention, interest, search, action, and sharing (Racherla and Friske, 2012). If consumers are attracted to a commodity, they show interest and desire to buy it and search relevant information online to decide whether to buy it or not. After the purchase, consumers post their feelings and experiences on social media or e-commerce websites. According to Schmitt et al. (2011), online WOM can be divided into organic WOM and firm-stimulated WOM. The former refers to consumers' spontaneous WOM, while the latter is WOM guided by enterprises. Online reviews are organic WOM, while online recommendations are marketer-stimulated WOM (Baum and Spann, 2014). Hussain et al. (2020) found that consumers' motivational involvement in electronic word of mouth for online information adoption is mediated by writers, motivations. The development of e-commerce and social media not only enriches the variety and connotation of products and services but also provides significant amounts of information that facilitates consumers' purchase decisions (Zhao et al., 2020). However, information overload has become a key issue in information search (Chen et al., 2009). Online recommendations cannot only reduce the information overload of consumers (Adomavicius et al., 2018) but also persuade consumers to consider or buy the recommended products (Ghose and Yang, 2009). However, in the internet-shopping environment, online reviews generated by previous customers supplement information on product and service providers (Zhang and Piramuthu, 2018), and consumers regard online reviews as a basis for deciding whether to buy the recommended products (Zhang et al., 2014). Choi and Lee (2017) suggested that user-generated content and marketer-generated content have different effects on consumer's trust, the former has more effect on cognitive trust while the latter influences emotional trust more. Therefore, online recommendations and reviews interactively influence consumers' purchase intention (Baum and Spann, 2014; Jabr and Zheng, 2014).

Information consistency measures the degree to which information from one source is consistent with information from another source, which is also known as information-source consistency (Walther et al., 2012). The high consistency of information from multiple sources makes information recipients feel that “the information should be credible” (Tormala and Clarkson, 2007), reducing their perception of uncertainty. However, in real life, information from multiple sources on the same product can either be consistent or inconsistent (Keh and Sun, 2018). For instance, reviews of a product can be positive in one source while negative in another source. The theory of cognitive dissonance states that an individual who finds information to be inconsistent feels psychologically conflicted and nervous and hopes to reduce this inconsistency by changing their attitude or behavior (Gawronski, 2012). Therefore, when information from two different sources (i.e., online recommendations and reviews) is inconsistent, consumers have difficulty in judging the authenticity of the information and the quality of products and thus, try to reduce the inconsistency by changing their attitude or behavior.

Looking at the product attributes discussed in online recommendations and reviews (De Maeyer, 2012), the types of information inconsistency between these two WOM vehicles were divided into vertical- and horizontal-attribute inconsistencies. Vertical product attributes refer to the attributes that consumers have clear and unified preference criteria for (e.g., battery life and volume for cellphone), and are regarded as quality attributes. Meanwhile, horizontal attributes refer to attributes that consumers have no unified preferences for and are often evaluated on different criteria based on consumers' personal preferences and feelings (e.g., color, appearance, and design of a cellphone). They are regarded as matching attributes (Wattal et al., 2009; Kwark et al., 2014). Since consumers have a unified preference criterion for vertical attributes, when information inconsistency (between online recommendations and reviews) mainly relates to vertical attributes, consumers' perceived inconsistency is higher. However, because consumers have no unified preference criterion for horizontal attributes, with higher information inconsistency, consumers perceive greater controversy of the product, from which they are more likely to generate a unique perception. The theory of the need for uniqueness argues that although individuals need to comply with popular social norms to avoid conflicts and win recognition, approval, or rewards from others, everyone has a desire to express their individuality and pursue differences (Franke and Schreier, 2008). The more unique a product is, the more it can satisfy the desires and demands of certain consumers for specific attributes. Once the specific needs of consumers are met, the products generate resonance. According to the theory of resonance in marketing, in a highly differentiated market, the higher the heterogeneity is, the higher the coincidence degree and intention to pay will be (Clemons et al., 2006). In other words, vertical-attribute inconsistency reduces consumers' purchase intention, while horizontal-attribute inconsistency may not. Therefore, we propose the following hypothesis:

Hypothesis 1: Compared with the impact of horizontal-attribute-information inconsistency, consumers' purchase intention is weaker when information on a product's vertical attribute in online recommendations and reviews is inconsistent.is was proposed.

### Perceived Uncertainty

Berger and Calabrese (1975) first put forward the uncertainty reduction theory (URT). They point out that in the early stage of interpersonal communication, due to lack of mutual understanding, there is often a certain degree of perceived uncertainty, which gradually decreases with deeper communication. Moreover, URT points out that, when consumers lack knowledge of a product, or they are unable to anticipate the outcome of consuming such a product, they actively search for other information to mitigate and eliminate the risks caused by uncertainty so as to obtain maximum value from the product (Chang et al., 2015).

Although consumers can seek and rely on information from multiple sources to reduce uncertainty (Chakravarty et al., 2010) perceived uncertainty may also originate from information asymmetry. When an individual perceives himself/herself as lacking sufficient information to make accurate predictions or he/she feels unable to discriminate between relevant and irrelevant data, he/she experiences uncertainty (Milliken, 1987). Shiu et al. (2011) argue that an individual, after knowing their needs, searches for relevant information about products or services to reduce uncertainty in a purchase decision. Particularly in the internet-shopping environment, perceived uncertainty mainly originates from two sources. One source is the information concerning products recommended online. Since consumers cannot conduct on-the-spot investigations of products and services, there is uncertainty in the authenticity of the information in recommendations (Wimmer and Yoon, 2017). The second source is information from other consumers' posts online. Due to anonymity in the online environment, online members have a weak-tie relationship; thus, it is impossible to accurately assess the validity of information shared by online members (Wang et al., 2018). Consumers have no way of distinguishing the intentions of those who make recommendations, as some of them exaggerate the benefits and quality of products for commercial interests, thereby making consumers feel uncertain about the information provided by online recommendations. Meanwhile, consumers feel uncertain about online reviews as they are unable to determine the authenticity of other people's reviews. Thus, it follows that when information from online recommendations and reviews is inconsistent, consumers inevitably perceive greater uncertainty. In the process of shopping, there are two main ways to eliminate perceived uncertainty: personal experience and information search (Weathers et al., 2007). Nevertheless, due to time and space constraints in the online shopping environment, consumers cannot “experience” the products/services in the pre-purchase process; instead, they can only reduce uncertainty through information search. However, when information from different sources is inconsistent, consumers perceive uncertainty.

When information from online recommendations is inconsistent with online reviews, consumers feel uncertain about the information's authenticity and validity (Wimmer and Yoon, 2017). Since consumers have a unified preference criterion for a product's vertical attributes, when information inconsistency between online recommendations and reviews mainly relate to vertical attributes, consumers cannot judge whether the information is relevant or not and cannot determine the quality of products, which leads to higher perceived uncertainty. However, since consumers have no unified preference criterion for horizontal attributes, with higher information inconsistency between online recommendations and reviews, consumers are more likely to develop unique perceptions and may attribute individual preferences or perceptions to cognitive disorders; thus, consumers' perception of uncertainty is lower. Perceived uncertainty makes it difficult for consumers to judge whether a commodity's attributes meet expectations, or even makes them lose the ability to identify the real attributes of a commodity (Dimoka et al., 2012; Hong and Pavlou, 2014), which affects their purchase intention. Existing literature has fully explored the impact of perceived uncertainty on consumer purchase behavior. Quintal et al. (2010) studied the impact of online tourism uncertainty on consumer behavior and found that uncertainty negatively affects consumers' attitudes and perceived behavior control. Previous studies also investigated the negative relationship between perceived uncertainty and online shopping intention (Pavlou et al., 2007; Yang et al., 2016). In the online shopping environment, it is found that perceived uncertainty originates from information inconsistency, which negatively affects purchase intentions. Thus, we propose the following hypothesis:

Hypothesis 2: Perceived uncertainty plays a mediating role between the type of information inconsistency and purchase intention. Specifically, compared with the effects of horizontal-attribute inconsistency, vertical-attribute inconsistency leads to higher perceived uncertainty, which further leads to lower purchase intention.

### Product Type

In considering whether consumers can perceive a product's quality before touching the product, Nelson (1970) proposed the SEC-product classification framework to classify a product as a search or experience product. Search products (e.g., cellphone, carpet) are those whose relevant attribute information can be easily obtained prior to use/purchase, while experience products (e.g., perfume, watch) are those whose relevant attribute information cannot be known until its trial or use.

Search products can be objectively evaluated and easily compared, while the evaluation and comparison of experience products are subjective and difficult (Huang et al., 2009). Consumers pay more attention to the reviews posted by previous consumers when they buy experience products, while they pay more attention to the quality information of the product when buying search products (Luan et al., 2016). Nelson (1970) argued that consumers' quality perception of search products involves objective attributes, while those of experience products depend more on subjective attributes (i.e., personal taste). Thus, for search products, objective reviews are more useful, while subjective reviews are more useful for experience products (Ghose and Yang, 2009). The evaluation of vertical attributes is often based on objective evaluations of a product's quality attributes, while the evaluation of horizontal attributes is often based on personalized evaluation of product-matching attributes (Wattal et al., 2009; Kwark et al., 2014). Thus, for search products, consumers are more inclined to consider evaluations of the product's vertical attributes. As such, when the information about a product's vertical attributes from online reviews is inconsistent with information from online recommendations, the impact on search products is greater. For experience products, consumers are more likely to adopt evaluations about a product's horizontal attributes. As such, when information about a product's horizontal attributes from online reviews is inconsistent with information from online recommendations, the impact on consumers' experience is greater. From existing literature, consumers show different emphasis on online WOM depending on the product type, indicating that the product type has a moderating effect between the type of information inconsistency and consumers' purchase intention (Chang et al., 2014). Thus, we propose the following hypotheses:

Hypothesis 3: The product's type has a moderating effect on the relationship between the type of information inconsistency and purchase intention.
Hypothesis 3a: For search products, vertical-attribute inconsistency has a more significant impact on purchase intention than that of horizontal-attribute inconsistency.
Hypothesis 3b: For experience products, horizontal-attribute inconsistency has a more significant impact on purchase intention than that of vertical-attribute inconsistency.

The perceived quality of search products is determined by objective attributes, while the perceived quality of experience products is determined more by subjective and personal experiences (Huang et al., 2009). For search products, consumers pay more attention to objective information and can make a basic judgment on product performance from objective information, while the corresponding value of subjective reviews is lower. For experience products, the subjective reviews of previous consumers are more significant (Mudambi and Schuff, 2010; Pan and Zhang, 2011). Consumers are more inclined to judge the quality of search products from evaluations of their vertical attributes. When information about vertical attributes from online reviews and recommendations is inconsistent, consumers experience greater uncertainty as they are unable to judge the quality of search products. Meanwhile, for experience products, when information about horizontal attributes is inconsistent, consumers experience greater uncertainty as they are unable to judge the quality of these products (Berger and Calabrese, 1975). Thus, we propose the following hypotheses:

Hypothesis 4: The product type mediates the effect of perceived uncertainty on the main effect.
Hypothesis 4a: For search products (compared with the effects of horizontal-attribute inconsistency), vertical-attribute inconsistency can enhance the mediating effect of perceived uncertainty on the main effect. Specifically, vertical-attribute inconsistency concerning search products (compared with horizontal-attribute inconsistency) leads to higher perceived uncertainty, which further leads to lower purchase intention.
Hypothesis 4b: For experience products (compared with the effects of vertical-attribute inconsistency), horizontal-attribute inconsistency can enhance the mediating effect of perceived uncertainty on the main effect. Specifically, horizontal-attribute inconsistency concerning experience products (compared with vertical-attribute inconsistency) leads to higher perceived uncertainty, which further leads to lower purchase intention.

**Figure 1:**
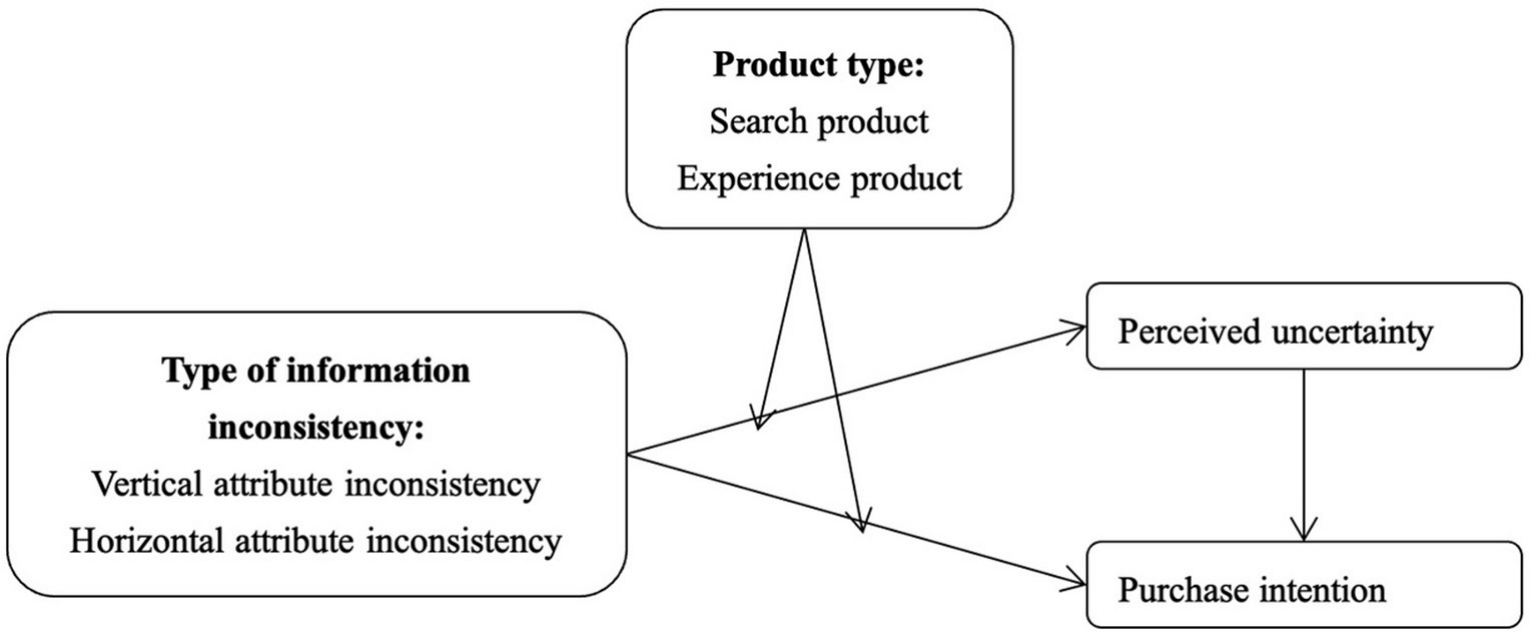
Conceptual Model.

The conceptual model is shown in Figure 1.

## Research methodology

This study selects people who have experience in using the online community platform as the survey object. As the original items were in English, we conducted a back translation to ensure translation validity. First, a researcher whose native language is Chinese translated the source items from English into Chinese. Next, another researcher independently translated these items back into English. Subsequently, the two researchers compared the two English versions and jointly revised the first Chinese version of the items. We then invited a panel of experts in the consumer behavior field to examine the face validity of the survey instrument. Based on their feedback, minor modifications were made to improve the comprehensiveness and user-friendliness of the measurement items. A pre-test of the survey instrument was conducted to conceptually validate the instrument. All items were measured on a 7-point Likert scale, which ranged from 1 (not agree at all) to 7 (absolutely agree).

### Pretest

First, based on the concept of product types, 10 professors and doctoral students with marketing major brainstormed and listed nine kinds of products, including sun-protective clothing, smartphone, laptop, book, movies, and catering (Chinese fondue). These products are either search or experience products and are highly recommended and reviewed online. A survey of 32 college students was conducted using a seven-point Likert scale, with the criterion of “whether one can perceive the quality of goods before purchase.” The product smartphone ranked first (with a score of 7.19), and catering ranked in the middle (5.78). Based on the definition of product types, the smartphone was selected as the search product and catering (Chinese fondue), the experience product.

Second, to determine the appropriate vertical and horizontal attributes of these products, product attributes that are considered when purchasing a smartphone and ordering Chinese fondue were divided into vertical and horizontal attributes and narrowed down to three as the focus of online reviews. We randomly selected 100 reviews of different brands and prices from Taobao and Dianping (a website that provides search and review services of restaurants and entertainment, among others, similar to Yelp.com). Each product's reviews were classified and integrated, and 15 product attributes of a smartphone, including operation system, pixel, tone quality, style, screen size, hand feel, endurance, color, thickness, material were confirmed. There were 10 product attributes of Chinese fondue, including environment, serving speed, service, taste, weight, type of dish, and decoration. Next, based on Sun (2011)'s definition of vertical and horizontal attributes, nine marketing postgraduates classified the different attributes of these three types of products. Then, three marketing postgraduates revised these classifications. Ten (six) vertical attributes and five (four) horizontal attributes of a smartphone (Chinese fondue) were determined. Subsequently, through random coding, a questionnaire was formed to rank the importance of the smartphone and Chinese fondue attributes (both vertical and horizontal). The three most important attributes of a smartphone and Chinese fondue were selected by 32 random subjects. Based on the frequency of selected attributes, we chose three representative vertical and horizontal attributes for each product (smartphone and Chinese fondue). For a smartphone, the vertical attributes were operation system (*n* = 23), pixel (*n* = 20), and endurance (*n* = 13), while the horizontal attributes were style (*n* = 21), hand feel (*n* = 19), and screen size (*n* = 15). For the product Chinese fondue, the vertical attributes were food (*n* = 21), environment (*n* = 18), and service (*n* = 16), while the horizontal attributes were taste (*n* = 27), weight (*n* = 19), and decoration style (*n* = 19).

### Experiment

#### Research Design

In this study, for collecting data, a quasi-experiment was used to simulate the real environment of online recommendations and reviews. To test the moderating role of product type, the experiment adopted a 2 (type of information inconsistency: vertical- vs. horizontal-attribute inconsistency)^*^2 (product type: search vs. experience product) between-subject design. There were four groups, with no <40 subjects in each group.

#### Procedure and Participants

##### Stimuli

To avoid the impact of the smartphone and Chinese fondue products' brands, prices, and other factors on the subjects' perception, the experiment only presented specific review-generated information points and added three typical specific reviews to facilitate the subjects' understanding. Simultaneously, the experiments also controlled for other information (e.g., additional reviews, reviewer, time, and amount) to eliminate the confounding effects. All reviews concealed marks that show the overall characteristics of reviews, such as the rate of good reviews, the number of reviews and others. Each group of recommended information contains only concept maps and two specific types of recommended content. Each group of reviewed information contains only six specific review points (including three vertical and three horizontal attributes that were obtained from the pretest) and three specific review content. The final stimuli information is presented in Appendix B.

##### Manipulation of Type of Information Inconsistency

The two types of control materials were in the picture way. The presentation and content of the pictures mimicked the real evaluation content form of online recommendations and reviews. For the content of specific reviews, the manipulation of online reviews was based on Chen and Xie (2008). First, based on the reviews of smartphones on Taobao and the reviews of Chinese fondue on Dianping, a typical positive review was selected. Second, according to the requirements of experimental manipulation, the positive adjectives in a review were replaced with their antonyms, and the interference of speech style and a number of words were controlled as much as possible. For example, “hand feel is good” was replaced by “hand feel is bad.” Vertical- (horizontal-) attribute inconsistency refers to inconsistent information between online recommendations and reviews that relates to vertical (horizontal) attributes. An example of vertical-attribute inconsistency is when online recommendations claim that a smartphone's operating system is powerful, while online reviews indicate that the operating system is average (or weak). Meanwhile, an example of horizontal-attribute inconsistency is when online recommendations claim that the feel (of a smartphone) is good, while online reviews indicate that the feel is ordinary. The controls used for Chinese fondue are the same as those for mobile phones.

##### Measurement of Mediating Variable

A seven-point Likert scale (1 = strongly disagree and 7 = strongly agree) was used. The scale of perceived uncertainty developed by Pavlou et al. (2007) includes four items that mainly measure consumer's perception of uncertainty from product information, product quality, online review authenticity, and online recommendation authenticity.

##### Measurement of Dependent Variable

A seven-point Likert scale (1 = strongly disagree and 7 = strongly agree) was used. Three items related to purchase intention were adopted from Gefen et al. (2003). The final survey questionnaire is presented in Appendix A.

We argued the rationality and feasibility of the experiment through a discussion with experts at a University in China. The experts put forward reasonable and feasible suggestions, such as avoiding uncontrollable factors (like the experiment's environment) and having all subjects answer questions in the network environment. All the scales were translated from an English scale. In the process of translation, a two-way translation method was adopted, and three graduate students proofread in multiple rounds to minimize semantic deviation in the process of translation.

##### Participant

The participants first tried a quasi-consumption exercise wherein they needed to purchase a smartphone/Chinese fondue online. They were interested in a product that was recommended by an online recommendation system. Next, they read reviews posted by previous consumers. Subsequently, as a manipulation test, the participants evaluated the product using the seven-point Likert scale in terms of product type, product attribute, and information consistency between online recommendations and reviews, and then evaluated the items related to perceived uncertainty and purchase intention. Finally, the participants provided their demographic information. A small gift was given to the subjects as a token of gratitude.

The participants in this experiment were all students at two universities in China. The respondents were in the age range of 18–28, 41.6% male, and mostly undergraduate and postgraduate students. Each group had at least 40 subjects, totaling 88 participants.

Consumers aged 18–30 years have gradually become the main consumer of the new generation. They are not only the main constituent users of the Internet but also have developed mature mobile shopping habits. This implies that the study of purchasing intention in the post-90s era (with regard to information inconsistency between online recommendations and reviews) is more relevant than that of previous periods.

## Results

### Manipulation Check

ANOVA was used to analyze the type of information inconsistency between online recommendations and reviews (vertical- vs. horizontal-attribute inconsistency). The results (Table 1) indicate that for a search product (smartphone), subjects in the vertical-attribute-inconsistency group perceived stronger vertical-attribute inconsistency than those in the horizontal-attribute-inconsistency group [M_vertical_ = 5.20, SD_vertical_ = 1.324 vs. M_horizontal_ = 2.86, SD_horizontal_ = 1.517; *F*_(1, 70)_ = 47.372, *P* < 0.001]. For an experience product (Chinese fondue), subjects in the vertical-attribute-inconsistency group perceived stronger vertical-attribute inconsistency than those in the horizontal-attribute-inconsistency group (M_vertical_ = 5.66, SD_vertical_ = 0.838 vs. M_horizontal_ = 2.71, SD_horizontal_ = 1.073; *F*_(1, 70)_ = 163.511, *P* < 0.001). Therefore, there is a significant difference between the type of information inconsistency, indicating that the manipulation of vertical-attribute inconsistency and horizontal-attribute inconsistency is successful.

**Table 1:** Manipulation check of type of information inconsistency.

**Product type**	**Type of information inconsistency**	**Mean**	**SD**	***F***	***P***
Search	Vertical attribute inconsistency	5.20	1.324	43.372	0.000
product	Horizontal attribute inconsistency	2.86	1.517		
Experience	Vertical attribute inconsistency	5.66	0.838	163.511	0.000
product	Horizontal attribute inconsistency	2.71	1.073		

### Main Effect

**Figure 2:**
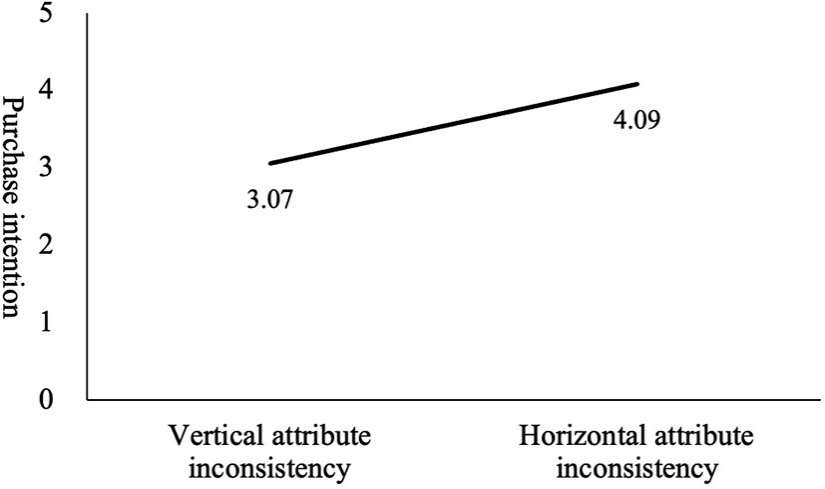
The effect of the type of information inconsistency on purchase intention.

The results of the reliability analysis of purchase intention are as follows. Cronbach's α (group 1) = 0.915 and Cronbach's α (group 2) = 0.913. After conducting a factor analysis rotation, a total of one factor was extracted, and the variance explanation degree was 85.25%. Next, a one-way ANOVA was used to test the main effect of the type of information inconsistency between online recommendations and reviews on purchase intention. First, the homogeneity test of variance shows that the variance was homogeneous (Levene = 0.465, *p* = 0.498). A further one-way ANOVA shows that the type of information inconsistency between online recommendations and reviews had a significant impact on purchase intention (M_vertical_ = 3.067, M_horizontal_ = 4.086, *F*_(1, 70)_ = 33.674, *P* < 0.05), and that when online recommendations and reviews had vertical-attribute inconsistency, consumers exhibited lower willingness to buy. Therefore, hypothesis 1 is supported. This indicates that when the information on vertical attributes of products in online recommendations and reviews is inconsistent consumers' purchase intention is weaker (compared with the case of horizontal attributes) (see Figure 2). According to the results of regression analysis, perceived uncertainty has a significant negative impact on purchase intention (β = −0.554, *P* < 0.001).

### Mediation Effect

A hierarchical regression model and the bootstrapping method were used in this study to test the hypotheses. The results of the hierarchical regression are shown in Table 2. Regarding the main effect, hypothesis 1 proposes that compared with the impact of horizontal-attribute inconsistency, consumers' purchase intention is weaker when the information on a product's vertical attribute in online recommendations and reviews is inconsistent. Results indicate that the type of information inconsistency has a significant negative impact on purchase intention (*r* = −0.236, *P* < 0.001). Therefore, supporting hypothesis 1.

**Table 2:** Results of hierarchical regression.

	**DV: perceived uncertainty**			**DV: purchase intention**			
	**Step 1**	**Step 2**	**Step 3**	**Step 1**	**Step 2**	**Step3**	**Step4**
Constant	5.321^***^	5.132^***^	4.869^***^	3.183^***^	6.197^***^	3.489^***^	5.315^***^
**Control variable**					
Gender	−0.198^***^	−0.138^***^	−0.001	−0.039	−0.402	−0.121^*^	−0.121^*^
Age	0.125	0.113	0.071	0.121	0.188	0.171	0.197^*^
Education	−0.080	−0.050	−0.045	−0.145	0.116	0.139	0.122
**Independent variable**						
Type of information inconsistency		0.219^***^	0.346^***^	−0.236^***^	−0.107^*^	−0.384^***^	−0.254^***^
**Mediator**							
Perceived uncertainty					−0.587^***^		−0.375^***^
**Moderating effect**							
Type of information inconsistency^*^ product type			0.354^***^			−0.412^***^	−0.280^***^
*F*-value	21.091	22.150	33.904	6.483	13.867	14.515	15.084
*R* square	0.318	0.396	0.559	0.161	0.341	0.351	0.405

*** *p < 0.001*,

* *p < 0.05*.

Following the analysis steps suggested by Baron and Kenny, this study used the transport hierarchical regression method to verify the mediating role of perceived uncertainty between the type of information inconsistency and purchase intention. The type of information inconsistency has a significant positive impact on perceived uncertainty (*r* = 0.219, *P* < 0.001) and a significant negative impact on purchase intention (*r* = −0.236, *P* < 0.001). After adding the mediating variable (perceived uncertainty), the influence of information inconsistency on purchase intention decreased significantly (*r* = −0.107, *P* < 0.05), but perceived uncertainty still has a significant negative impact on purchase intention (*r* = −0.587, *P* < 0.001), indicating that perceived uncertainty plays a mediating role between information inconsistency and purchase intention. The results show that perceived uncertainty plays a mediating role between the type of information inconsistency and purchase intention. Specifically, compared with the effects of horizontal-attribute inconsistency, vertical-attribute inconsistency leads to higher perceived uncertainty, which further leads to lower purchase intention. Thus, hypothesis 2 is supported.

### Moderating Effect

**Figure 3:**
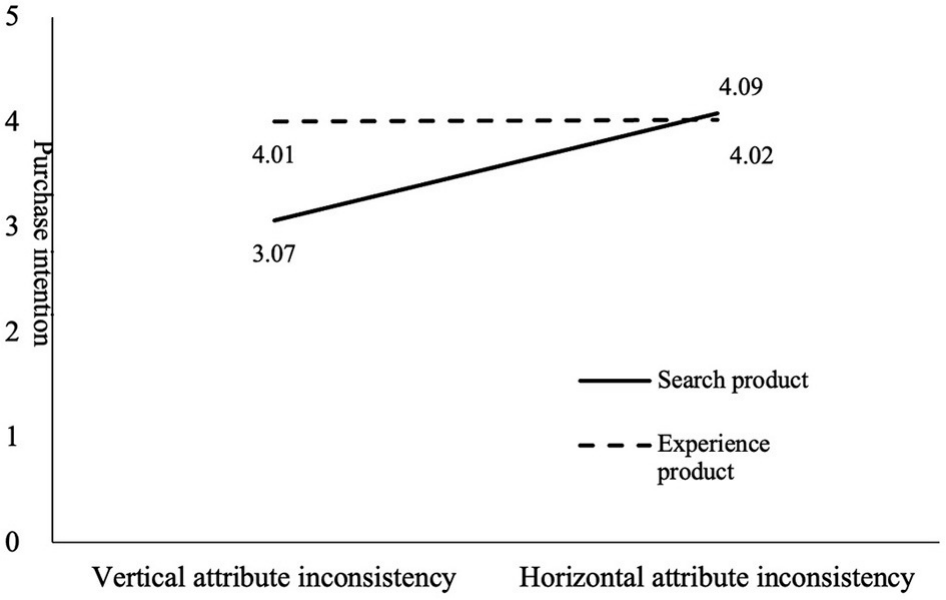
The effect of product type on the main effect.

Hypothesis 3 proposes that the product type has a moderating effect on the relationship between the type of information inconsistency and purchase intention. To eliminate collinearity, the independent and moderating variables were standardized when the product terms of the independent and moderating variables were constructed. We set the independent variables vertical-attribute inconsistency to 1 and the horizontal-attribute inconsistency to −1; and the moderating variables search product to 1 and experience product to −1. From Table 2, we can see that the interaction between the type of information inconsistency and product type has a significant negative effect on purchase intention (*r* = −0.412, *P* < 0.001), which indicates that the product type can enhance the relationship between the type of information inconsistency and purchase intention, thus supporting H3. The sub-hypotheses of hypothesis 3 were tested using ANOVA. The results show that for search products, vertical-attribute inconsistency has a more significant impact on purchase intention than that of horizontal-attribute inconsistency, and hypothesis 3a is supported (M_vertical_ = 3.067, M_horizontal_ = 4.086, *r* = 2.098, *P* < 0.001). But for experience products, horizontal-attribute inconsistency has a not significant impact on purchase intention, and hypothesis 3b is not supported (M_vertical_ = 4.010, M_horizontal_ = 4.019, *r* = 2.098, *P* < 0.001, see Table 3 and Figure 3). Meanwhile, gender has an impact on perceived uncertainty and purchase intention as there are differences in the search-related endurance and purchase desires of men and women.

**Table 3:** Test of product type on main effect.

**Product types**	**Levene (*p*)**	**M_**vertical**_**	**M_**horizontal**_**	***F***	***P***
Search product	2.098 (0.152)	3.067	4.086	33.674	0.000
Experience product	1.853 (0.178)	4.010	4.019	0.009	0.926

*Levene is value of homogeneity test*.

### Moderated Mediation Effect

**Figure 4:**
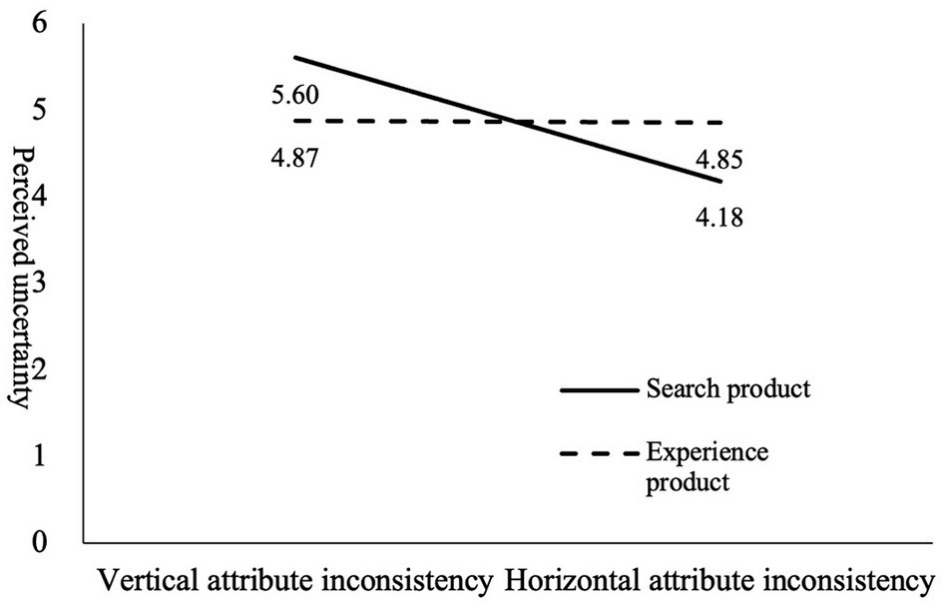
The effect of product type on mediation effect.

Hypothesis 4 proposes that the product type serves as a moderator in the mediating effect of perceived uncertainty on the main effect. We conducted a bootstrap analysis for estimating the moderated mediation effect on 5,000 samples (SPSS Macro PROCESS, Model 8), using the type of information inconsistency as the independent variable, perceived uncertainty concern as the mediator, product type as the moderator, and purchase intention as the dependent variable. The results show that the product type has a significant moderating effect on the main effect [index = −0.2513, 95% CI = (−0.4209, −0.0906)]. Furthermore, the sub-hypotheses of hypothesis 4 were tested using ANOVA. The results show that vertical-attribute inconsistency concerning search products (compared with horizontal-attribute inconsistency) leads to higher perceived uncertainty, which further leads to lower purchase intention, the hypothesis 4a is supported (M_vertical_ = 5.600, M_horizontal_ = 4.179, *r* = 1.204, *P* < 0.001). And horizontal-attribute inconsistency concerning experience products (compared with vertical-attribute inconsistency) has no significant effect on perceived uncertainty and purchase intention (M_vertical_ = 4.871, M_horizontal_ = 4.850, *r* = 0.592, *P* > 0.1), the hypothesis 4b is not supported (see Table 4 and Figure 4). This may be because, for experiential products, consumers pay more attention to the impact of vertical-attribute inconsistency on purchase intention, so there is no significant impact.

**Table 4:** Test of product type on mediation effect.

**Product types**	**Levene (*p*)**	**M_**vertical**_**	**M_**horizontal**_**	***F***	***P***
Search product	1.204 (0.276)	5.600	4.179	226.902	0.000
Experience product	0.592 (0.444)	4.871	4.850	0.025	0.875

*Levene is value of homogeneity test*.

## Discussion

The results show that the type of information inconsistency between online recommendations and reviews affects consumers' purchase intention. Specifically, compared with the effect of horizontal-attribute inconsistency, when information on a product's vertical attribute is inconsistent, consumers' purchase intention becomes weaker, that is, vertical-attribute inconsistency between online recommendations and reviews has a greater negative impact on consumers' purchase intention. Meanwhile, hypothesis 2 is also supported, indicating that perceived uncertainty plays a mediating role in the influence of the type of information inconsistency between online recommendations and reviews on consumers' purchase intention.

The results also support hypothesis 3a, indicating that the product type (search or experience) has a moderating effect on the main effect, implying that the higher the information inconsistency is in the vertical/horizontal attributes of search products, the lower the purchase intention will be. Specifically, for search product (compared with the impact on experience product), the impact of vertical-attribute inconsistency on the main effect is more significant. However, hypothesis 3b is not supported in this experiment. The results show that for experience products, there is no significant difference between the impacts of vertical- and horizontal-attribute inconsistencies on the purchase intention. Furthermore, the results support hypothesis 4a. Specifically, compared with the case of experience products, vertical-attribute inconsistency can enhance the moderating effect of perceived uncertainty on the main effect of search products. However, the results do not support hypothesis 4b.

## Conclusions

### Research Results

This study explored the interaction of the type of information inconsistency (vertical- vs. horizontal-attribute inconsistency) and the product type (search vs. experience products) on perceived uncertainty and purchase intention. In this study, 2 (vertical- vs. horizontal-attribute inconsistency) × 2 (product type: search vs. experience products) analyses were conducted to test the main effect and the boundary condition of the moderating role of product type. Moreover, this study divided the type of information inconsistency between online recommendations and reviews into vertical- and horizontal-attribute inconsistencies. The main conclusion of this study is that for search products, vertical-attribute inconsistency leads to a higher perceived uncertainty and lower purchase intention. However, for experience products, there is no significant difference in the impact of vertical- and horizontal- attribute inconsistencies on purchase intention.

### Contributions and Implications

This article explored the interaction of online recommendations and reviews on purchase intention and revealed the underlying mechanisms and the moderating role of the product type on such mechanisms. The study has certain theoretical contributions, as well as managerial implications. It not only enriches the related research on online WOM but also provides targeted guidance for enterprises that carry out online WOM management.

#### Theoretical Contributions

This study explored the effect of the type of information inconsistency between online recommendations and reviews on purchase intention, as well as considered the moderating role of product type. The theoretical contributions are mainly embodied in three aspects as follows.

First, although the two main forms of online WOM, online recommendations and reviews (Schmitt et al., 2011), have been extensively studied in the literature (Seiler et al., 2017; Wang et al., 2018), most of the previous studies investigated them independently (Utz et al., 2012; Elwalda et al., 2016; Hong et al., 2017). Moreover, existing research on the interaction between the two vehicles of online WOM simply regarded them as different sources of information for studying information consistency and consumers' willingness to purchase (Baum and Spann, 2014; Jabr and Zheng, 2014). Moreover, these previous studies did not thoroughly examine the effect of the type of information inconsistency between these two types of online WOMs on perceived uncertainty and purchase intention, nor tested the moderating role of the product type. Meanwhile, this present study noted that online recommendations and reviews often interact to influence consumers' perceived uncertainty and purchase intention. When information from online recommendations is inconsistent with that of online reviews, consumers neither judge the quality of the product nor make a purchase decision. Furthermore, for different types of products (Keh and Sun, 2018), different types of information inconsistency have different impacts on consumers' intention to purchase.

Second, from the perspective of product attribute, the type of information inconsistency between online recommendations and reviews were divided into vertical- and horizontal-attribute inconsistencies in this study. De Maeyer (2012) conducted research on the product attributes involved in the review content when researching online reviews. Meanwhile, this study explored product attributes in light of specific aspects of information inconsistency between online recommendations and reviews, thereby expanding the theory of consumer decision-making behavior in the online shopping environment.

Third, this study introduced product type as a moderating variable to study the impact of WOM inconsistency on consumer behavior for different types of products. The classical theory of product type was introduced (Nelson, 1970; Girard and Dion, 2010; Sun, 2011), enriching the generalization and applicability of the research.

Finally, this study makes comprehensive use of ANOVA and regression analysis, which is original in research methods. In this study, types of information inconsistency and product types are used as category variables, and used experimental method in the analysis. This study also confirmed the mediating role of perceived uncertainty and the moderating role of product types, and explored the influencing factors of online reviews on consumers' purchase intention. This study provides methodological support for a better understanding of the dark side of consumers' purchase intention online reviews in e-commerce.

#### Managerial Implications

Based on the current situation of consumer consumption, this study provides an analytical framework for important decision-making issues encountered by companies in the process of eWOM management and has important practical implications. The findings of this study are useful for companies to more appropriate to distinguish the role of negative WOM and specifically manage the online recommendation system and online review information.

First, this study classified information inconsistency between online recommendations and reviews. The results show that the company's online recommendation can effectively attract consumers, and online reviews can effectively facilitate consumers' decision-making. Knowing how to manage online reviews and how to promote network-traffic conversion are extremely important for companies. This study found that for search products, vertical-attribute inconsistency between online recommendations and reviews has greater negative impact on purchase intention, which may lead to lower purchase intention. Thus, companies should improve the vertical attributes of search products. Improving search products' horizontal attributes by guaranteeing the quality of the products' vertical attributes, guiding consumers to conduct online reviews on the vertical attributes of these search products, and increasing the volume of more realistic and detailed vertical-attribute-related reviews to improve consumers' perception of the quality of search products can all help reduce consumers' perception of uncertainty.

Second, this study found that for experience products, vertical- and horizontal-attribute inconsistency between online recommendations and reviews have no significant difference with regard to their impact on consumers' purchase intention. This is mainly because the quality or performance information of experience products can only be obtained by purchasing and using these products. This emphasizes the significance of users' personal experience, and this experience often varies from person to person. This study found that when information on products' vertical attributes in online recommendations and reviews are inconsistent, no significant difference exists in the influence of the horizontal attribute on consumers' purchase intention, implying that these consumers rely upon and value their experiences highly. As such, companies should adopt various forms of customer engagement, such as product trials and different modes of offline experience, to increase users' experience and further enhance their purchase intention.

Third, the findings of this study can help companies manage their online recommendations and reviews in two ways that could in turn increase sales. On the one hand, companies can use online recommendations to guide consumers to conduct online reviews. On the other hand, online reviews can further improve online recommendations. Promoting the consistency between online recommendations and reviews, effectively using online recommendations to attract more consumers, and using online reviews to transform network traffic are ways by which companies can increase their sales.

## Limitations and Future Research

Aside from the conclusions discussed above, this study also has theoretical contributions and managerial implications, as well as limitations. First, the sample size was not comprehensive and the study participants were concentrated to post-90s students. Future research can expand the sample coverage and include other age groups. Second, this study only considered two types of information inconsistency between online recommendations and reviews in terms of product attributes. Future research may consider other types of information inconsistency. Third, this study explored the relationship between information (in)consistency and perceived uncertainty. Future research may explore more mediators. For example, Shen et al. (2018) examined the effect of content consistency and process consistency on perceived fluency, as well as omnichannel service usage.

## Data Availability Statement

The raw data supporting the conclusions of this article will be made available by the authors, without undue reservation.

## Ethics Statement

The studies involving human participants were reviewed and approved by the School of Business, Changshu Institute of Technology. Written informed consent for participation was not required for this study in accordance with the national legislation and the institutional requirements.

## Author Contributions

JW designed the study and drafted the initial manuscript. YG and XF collected the data, performed statistical analysis, and drafted the initial manuscript. XS contributed to the revised manuscript. All authors discussed the results and contributed to the final manuscript.

## Funding

This paper was supported by the China Postdoctoral Science Foundation (2021M690654) and Jiangsu University Philosophy and Social Science Research Major Project (2021SJZDA033).

## Conflict of Interest

The authors declare that the research was conducted in the absence of any commercial or financial relationships that could be construed as a potential conflict of interest.

## Publisher's Note

All claims expressed in this article are solely those of the authors and do not necessarily represent those of their affiliated organizations, or those of the publisher, the editors and the reviewers. Any product that may be evaluated in this article, or claim that may be made by its manufacturer, is not guaranteed or endorsed by the publisher.

## Appendix A

**Table 1:**

**Construct**	**Measurement item**	**References**
**Perceived uncertainty**	**1. I feel that buying products through online reviews involves a high degree of uncertainty**	**Pavlou et al., 2007**
	**2. I feel the uncertainty associated with information inconsistency from online reviews is high**.	
	**3. I am exposed to many transaction uncertainties if I buying products through online review**.	
	**4. There is a high degree of product uncertainty (i.e., the product you receive may not be exactly what you want) when purchasing online products through online reviews**.	
**Purchase intention**	**1. I believe this product is my first choice**.	**Gefen et al., 2003**
	**2. I will choose this product next time when I want to buy**.	
	**3. I will speak positively about this product to others**.	

## References

[B1] Adomavicius G. Bockstedt J. C. Curley S. P. Zhang J. (2018). Effects of online recommendations on consumers' willingness to pay. Inf. Syst. Res. 29, 84–102. 10.1287/isre.2017.0703

[B2] Ajzen I. (1991). The theory of planned behavior. Org. Behav. Human Decis. Proc. 50, 179–211 10.1016/0749-5978(91)90020-T

[B3] Azer J. Alexander M. (2020). Direct and indirect negatively valenced engagement behavior. J. Serv. Market. 34, 967–981. 10.1108/JSM-08-2019-0296

[B4] Baum D. Spann M. (2014). The interplay between online consumer reviews and recommender systems: an experimental analysis. Int. J. Electron. Commer. 19, 129–161. 10.2753/JEC1086-4415190104

[B5] Berger C. R. Calabrese R. J. (1975). Some explorations in initial interaction and beyond: toward a developmental theory of interpersonal communication. Hum. Commun. Res. 1, 99–112. 10.1111/j.1468-2958.1975.tb00258.x

[B6] Chakravarty A. Liu Y. Mazumdar T. (2010). The differential effects of online word-of-mouth and critics' reviews on pre-release movie evaluation. J. Interact. Mark. 24, 185–197. 10.1016/j.intmar.2010.04.001

[B7] Chang H. H. Fang P. W. Huang C. H. (2015). The impact of on-line consumer reviews on value perception: the dual-process theory and uncertainty reduction. J. Organ. End User Comput. 27, 32–57. 10.4018/joeuc.2015040102

[B8] Chang S.-T. Lin T. M. Y. Luarn P. (2014). The effects of word-of-mouth consistency on persuasiveness. Can. J. Adm. Sci. 31, 128–141. 10.1002/cjas.1279

[B9] Chen M.-J. Farn C.-K. (2020). Examining the influence of emotional expressions in online consumer reviews on perceived helpfulness. Inf. Process. Manage. 57:102266. 10.1016/j.ipm.2020.102266

[B10] Chen Y. Xie J. (2008). Online consumer review: Word-of-mouth as a news element of marketing communication mix. Manage. Sci. 54, 477–491. 10.1287/mnsc.1070.0810

[B11] Chen Y.-C. Shang R.-A. Kao C.-Y. (2009). The effects of information overload on consumers' subjective state towards buying decision in the internet shopping environment. Electron. Commer. Res. Appl. 8, 48–58. 10.1016/j.elerap.2008.09.001

[B12] Cheung C. M. K. Lee M. K. O. Rabjohn N. (2008). The impact of electronic word-of-mouth - the adoption of online opinions in online customer communities. Internet Res. 18, 229–247. 10.1108/10662240810883290

[B13] Choi B. Lee I. (2017). Trust in open versus closed social media: the relative influence of user- and marketer-generated content in social network services on customer trust. Telemat. Inf. 34, 550–559. 10.1016/j.tele.2016.11.005

[B14] Choi J. Yoon J. Chung J. Coh B.-Y. Lee J.-M. (2020). Social media analytics and business intelligence research: a systematic review. Inf. Process. Manage. 57:102279. 10.1016/j.ipm.2020.102279

[B15] Clemons E. K. Gao G. G. Hitt L. M. (2006). When online reviews meet hyperdifferentiation: a study of the craft beer industry. J. Manage. Inform. Syst. 23, 149–171. 10.2753/MIS0742-1222230207

[B16] De Maeyer P. (2012). Impact of online consumer reviews on sales and price strategies: a review and directions for future research. J. Prod. Brand Manag. 21, 132–139. 10.1108/10610421211215599

[B17] Dimoka A. Hong Y. Pavlou P. A. (2012). On product uncertainty in online markets: theory and evidence. MIS Q. 36, 395–426. 10.2307/41703461

[B18] Eelen J. Ozturan P. Verlegh P. W. J. (2017). The differential impact of brand loyalty on traditional and online word of mouth: the moderating roles of self-brand connection and the desire to help the brand. Int. J. Res. Mark. 34, 872–891. 10.1016/j.ijresmar.2017.08.002

[B19] Elwalda A. Lue K. Ali M. (2016). Perceived derived attributes of online customer reviews. Comput. Hum. Behav. 56, 306–319. 10.1016/j.chb.2015.11.051

[B20] Eslami S. Ghasemaghaei M. (2018). Effects of online review positiveness and review score inconsistency on sales: a comparison by product involvement. J. Retai. Consum. Serv. 45, 74–80. 10.1016/j.jretconser.2018.08.003

[B21] Fishbein M. Ajzen I. (1975). Belief, Attitude, Intention, and Behavior: An Introduction to Theory and Research. Reading, MA: Addison-Wesley, 53.

[B22] Franke N. Schreier M. (2008). Product uniqueness as a driver of customer utility in mass customization. Mark. Lett. 19, 93–107. 10.1007/s11002-007-9029-7

[B23] Gawronski B. (2012). Back to the future of dissonance theory: cognitive consistency as a core motive. Soc. Cogn. 30, 652–668. 10.1521/soco.2012.30.6.652

[B24] Gefen D. Karahanna E. Straub D. W. (2003). Trust and TAM in online shopping: an integrated model. MIS Q. 27, 51–90. 10.2307/30036519

[B25] Ghose A. Yang S. (2009). An empirical analysis of search engine advertising: sponsored search in electronic markets. Manage. Sci. 55, 1605–1622. 10.1287/mnsc.1090.1054

[B26] Girard T. Dion P. (2010). Validating the search, experience, and credence product classification framework. J. Bus. Res. 63, 1079–1087. 10.1016/j.jbusres.2008.12.011

[B27] Hall J. A. (2018). When is social media use social interaction? Defining mediated social interaction. New Media Soc. 20, 162–179. 10.1177/1461444816660782

[B28] Hong Y. Pavlou P. A. (2014). Product fit uncertainty in online markets: nature, effects, and antecedents. Inf. Syst. Res. 25, 328–344. 10.1287/isre.2014.0520

[B29] Hong Y. Pavlou P. A. Shi N. Wang K. (2017). On the role of fairness and social distance in designing in effective social referral systems. MIS Q. 41, 787–809. 10.25300/MISQ/2017/41.3.06

[B30] Huang A. H. Chen K. Yen D. C. Tran T. P. (2015). A study of factors that contribute to online review helpfulness. Comput. Hum. Behav. 48, 17–27. 10.1016/j.chb.2015.01.010

[B31] Huang P. Lurie N. H. Mitra S. (2009). Searching for experience on the web: an empirical examination of consumer behavior for search and experience goods. J. Mark. 73, 55–69. 10.1509/jmkg.73.2.55

[B32] Hussain S. Song X. Niu B. (2020). Consumers' motivational involvement in eWOM for information adoption: the mediating role of organizational motives. Front. Psychol. 10, 1–13. 10.3389/fpsyg.2019.03055 32038413 PMC6985457

[B33] Jabr W. Zheng Z. (2014). Know yourself and know your enemy: An analysis of firm recommendations and consumer reviews in a competitive environment. MIS Q. 38, 635–U423. 10.25300/MISQ/2014/38.3.01

[B34] Keh H. T. Sun J. (2018). The Differential effects of online peer review and expert review on service evaluations: the roles of confidence and information convergence. J. Serv. Res. 21, 474–489. 10.1177/1094670518779456

[B35] Kim E. L. Tanford S. (2019). Simultaneous effects of multiple cues in restaurant reviews. J. Serv. Mark. 33, 521–531. 10.1108/JSM-06-2018-0188

[B36] Kwark Y. Chen J. Raghunathan S. (2014). Online product reviews: implications for retailers and competing manufacturers. Inf. Syst. Res. 25, 93–110. 10.1287/isre.2013.0511

[B37] Lantzy S. Anderson D. (2020). Can consumers use online reviews to avoid unsuitable doctors? Evidence from RateMDs.com and the Federation of State Medical Boards. Decis. Sci. 51, 962–984. 10.1111/deci.12398

[B38] Liang Y. (2016). Reading to make a decision or to reduce cognitive dissonance? The effect of selecting and reading online reviews from a post-decision context. Comput. Hum. Behav. 64, 463–471. 10.1016/j.chb.2016.07.016

[B39] Luan J. Yao Z. Zhao F. T. Liu H. (2016). Search product and experience product online reviews: an eye tracking study on consumers' review search behavior. Comput. Hum. Behav. 65, 420–430. 10.1016/j.chb.2016.08.037

[B40] Ma Y. Chen G. Wei Q. (2017). Finding users preferences from large-scale online reviews for personalized recommendation. Electron. Commer. Res. 17, 3–29. 10.1007/s10660-016-9240-9

[B41] Milliken F. J. (1987). Three types of perceived uncertainty about the environment: state, effect, and response uncertainty. Acad. Manage. Rev. 12, 133–143. 10.5465/amr.1987.4306502

[B42] Mudambi S. M. Schuff D. (2010). What makes a helpful online review? A study of coustomer reviews on amazon.com. MIS Q. 34, 185–200. 10.2307/20721420

[B43] Nelson P. (1970). Information and consumer behavior. J. Polit. Econ. 78, 311–329. 10.1086/259630

[B44] Pan Y. Zhang J. Q. (2011). Born unequal: a study of the helpfulness of user-generated product reviews. J. Retail. 87, 598–612. 10.1016/j.jretai.2011.05.002

[B45] Pavlou P. A. Liang H. Xue Y. (2007). Understanding and mitigating uncertainty in online exchange relationships: a principal-agent perspective. MIS Q. 31, 105–136. 10.2307/25148783

[B46] Quaschning S. Pandelaere M. Vermeir I. (2015). When consistency matters: the effect of valence consistency on review helpfulness. J. Comput.-Mediat. Commun. 20, 136–152. 10.1111/jcc4.12106

[B47] Quintal V. A. Lee J. A. Soutar G. N. (2010). Risk, uncertainty and the theory of planned behavior: a tourism example. Tourism Manage. 31, 797–805. 10.1016/j.tourman.2009.08.006

[B48] Racherla P. Friske W. (2012). Perceived 'usefulness' of online consumer reviews: an exploratory investigation across three services categories. Electron. Commer. Res. Appl. 11, 548–559. 10.1016/j.elerap.2012.06.003

[B49] Rosario A. B. Sotgiu F. De Valck K. Bijmolt T. H. A. (2016). The effect of electronic word of mouth on sales: a meta-analytic review of platform, product, and metric factors. J. Mark. Res. 53, 297–318. 10.1509/jmr.14.0380

[B50] Schmitt P. Skiera B. Van den Bulte C. (2011). Referral programs and customer value. J. Mark. 75, 46–59. 10.1509/jm.75.1.46

[B51] Seiler S. Yao S. Wang W. (2017). Does online word of mouth increase demand? (and how?) Evidence from a natural experiment. Mark. Sci. 36, 838–861. 10.1287/mksc.2017.1045

[B52] Shen X. L. Li Y. J. Sun H. Q. Wang N. (2018). Channel integration quality, perceived fluency and omnichannel service usage: The moderating roles of internal and external usage experience. Decis. Support Syst. 109, 61–73. 10.1016/j.dss.2018.01.006

[B53] Shiu E. M. K. Walsh G. Hassan L. M. Shaw D. (2011). Consumer uncertainty, revisited. Psychol. Mark. 28, 584–607. 10.1002/mar.20402

[B54] Sun M. (2011). Disclosing multiple product attributes. J. Econ. Manage. Strategy 20, 195–224. 10.1111/j.1530-9134.2010.00287.x

[B55] Tormala Z. L. Clarkson J. J. (2007). Assimilation and contrast in persuasion: the effects of source credibility in multiple message situations. Pers. Soc. Psychol. Bull. 33, 559–571. 10.1177/0146167206296955 17363764

[B56] Utz S. Kerkhof P. van den Bos J. (2012). Consumers rule: how consumer reviews influence perceived trustworthiness of online stores. Electron. Commer. Res. Appl. 11, 49–58. 10.1016/j.elerap.2011.07.010

[B57] Walther J. B. Liang Y. Ganster T. Wohn D. Y. Emington J. (2012). Online reviews, helpfulness ratings, and consumer attitudes: an extension of congruity theory to multiple sources in web 2.0. J. Comput.-Mediat. Commun. 18, 97–112. 10.1111/j.1083-6101.2012.01595.x

[B58] Wang J.-J. Wang L.-Y. Wang M.-M. (2018). Understanding the effects of eWOM social ties on purchase intentions: a moderated mediation investigation. Electron. Commer. Res. Appl. 28, 54–62. 10.1016/j.elerap.2018.01.011

[B59] Wattal S. Telang R. Mukhpadhyay T. (2009). Information personalization in a two-dimensional product differentiation model. J. Manage. Inform. Syst. 26, 69–95. 10.2753/MIS0742-1222260204

[B60] Weathers D. Sharma S. Wood S. L. (2007). Effects of online communication practices on consumer perceptions of performance uncertainty for search and experience goods. J. Retail. 83, 393–401. 10.1016/j.jretai.2007.03.009

[B61] Wimmer H. Yoon V. Y. (2017). Counterfeit product detection: bridging the gap between design science and behavioral science in information systems research. Decis. Support Syst. 104, 1–12. 10.1016/j.dss.2017.09.005

[B62] Wu T.-Y. Lin C. A. (2017). Predicting the effects of eWOM and online brand messaging: source trust, bandwagon effect and innovation adoption factors. Telemat. Inf. 34, 470–480. 10.1016/j.tele.2016.08.001

[B63] Yan Q. Wu S. Wang L. Wu P. Chen H. Wei G. (2016). E-WOM from e-commerce websites and social media: which will consumers adopt? Electron. Commer. Res. Appl. 17, 62–73. 10.1016/j.elerap.2016.03.004

[B64] Yang J. Sarathy R. Lee J. (2016). The effect of product review balance and volume on online Shoppers' risk perception and purchase intention. Decis. Support Syst. 89, 66–76. 10.1016/j.dss.2016.06.009

[B65] Zhang J. H. Piramuthu S. (2018). Product recommendation with latent review topics. Inf. Syst. Front. 20, 617–625. 10.1007/s10796-016-9697-z

[B66] Zhang K. Z. K. Zhao S. J. Cheung C. M. K. Lee M. K. O. (2014). Examining the influence of online reviews on consumers' decision-making: a heuristic-systematic model. Decis. Support Syst. 67, 78–89. 10.1016/j.dss.2014.08.005

[B67] Zhao Y. Wang L. Tang H. J. Zhang Y. M. (2020). Electronic word-of-mouth and consumer purchase intentions in social e-commerce. Electron. Comm. Res. Appl. 41:100980. 10.1016/j.elerap.2020.100980

[B68] Zheng X. Hong Y. Ren X. Cao J. Yang S. (2018). Information inconsistencies in multi-dimensional rating systems. In International Conference on Information Systems 2018, ICIS 2018 (International Conference on Information Systems 2018, ICIS 2018). Association for Information Systems.

